# Effectiveness and tolerability of migalastat in adult Fabry disease: A single regional centre experience

**DOI:** 10.1016/j.ymgmr.2026.101316

**Published:** 2026-05-11

**Authors:** Eamon P. McCarron, Rajkumar Chinnadurai, Karolina M. Stepien, John Bassett, Jonathan Meyer, Reena Sharma, Peter Woolfson, Ana Jovanovic

**Affiliations:** a Adult Inherited Metabolic Diseases, Salford Care Organisation, Northern Care Alliance (NCA) National Health Service (NHS) Foundation Trust, Stott Lane, Salford, UK; b Adult Inherited Metabolic Disorders, Sheffield NHS Foundation Trust, Herries Drive, Sheffield, UK; c Faculty of Health, University of Sheffield, Sheffield, UK; d Department of Renal Medicine, Salford Care Organisation, NCA NHS Foundation Trust, Salford, UK; e Division of Cardiovascular Sciences, University of Manchester, Manchester, UK; f Department of Cardiology, Salford Care Organisation, NCA NHS Foundation Trust, Salford, UK; g The School of Medicine, Manchester Academic Health Sciences Centre, Manchester University, UK

**Keywords:** Fabry disease, Alpha-galactosidase, Migalastat, Globotriaosylsphingosine

## Abstract

**Background:**

Fabry disease (FD) is an X-linked lysosomal storage disorder caused by deficient α-galactosidase A (α-Gal) activity, leading to progressive renal, cardiac, and cerebrovascular involvement. Migalastat, an oral pharmacological chaperone, is indicated for patients with amenable *GLA* variants. Although efficacy has been demonstrated in clinical trials, long-term real-world data remain necessary, particularly in mixed groups of treatment-naïve and ERT-switch patients and genotype-phenotype subgroups.

**Methods:**

Eighty-seven adults with FD (55 males, 32 females) carrying *GLA* variants were retrospectively analysed at a single regional centre. Patients received migalastat as treatment naïve (TN) (*n* = 48) or treatment switch (TS) (*n* = 39). Demographic, genotypic, biochemical, renal, and cardiac parameters, and clinical outcomes were evaluated.

**Results:**

Variants were predominantly late-onset (67%), most commonly c.644 A > G (p.Asn215Ser)/p.N215S; 14% were classical and 19% unclassified. Plasma globotriaosylsphingosine (lyso-Gb3) declined in both groups (TN: 5.1 (3.7–7.9) to 2.2 (1.3–3.6) ng/mL; TS: 7.2 (3.8–13.0) to 3.3 (1.4–6.7) ng/mL). Renal function remained above clinically relevant thresholds in most patients, with a modest reduction in the proportion maintaining eGFR >90 mL/min/1.73 m^2^. Blood pressure, and echocardiographic measurements were largely stable; however, a significant reduction (*P* = 0.009) in left ventricular mass index (LVMI) was observed in TS patients when restricting analysis to variants classified as amenable according to the Galafold® amenability table (United Kingdom (UK), 2024 version). In TS patients, QTc increased significantly (*P* < 0.001) but remained within normal or only mildly prolonged limits. Sex-stratified analyses showed higher LVMI in males than females at baseline, with non-significant reductions in both groups over time. The incidence of non-fatal cardiovascular events was 81 and 43.5 per 1000 person-years in TN and TS groups, respectively. Six male patients (6.9%) experienced adverse events; five discontinued treatment, and one Fabry-related death occurred in the TN group.

**Conclusion:**

Migalastat was well tolerated and maintained stable renal and cardiac parameters with plasma lyso-Gb3 improvement. However, a subset of patients showed progression or intolerance, underscoring that variant amenability alone may not predict clinical benefit.

## Highlights

• Migalastat demonstrated sustained renal and cardiac stability in real-world Fabry disease.
• Plasma lyso-Gb3 levels decreased in both treatment-naïve and switch groups.
• Left ventricular mass index improved in treatment-switch patients with amenable variants.
• Clinical progression or intolerance occurred in a minority, indicating amenability alone may not ensure benefit.

## 1 Introduction

Fabry disease (FD, OMIM #301500) is a rare X-linked lysosomal storage disorder caused by mutations in the *GLA* gene, resulting in deficient α-galactosidase A (α-Gal) activity and accumulation of globotriaosylceramide (Gb3) and related glycosphingolipids in multiple tissues. [1], [2] FD encompasses a spectrum of phenotypes; patients with late-onset variants often present in adulthood with progressive renal dysfunction, cardiomyopathy, cerebrovascular disease, and gastrointestinal symptoms. [3] Many experience significant diagnostic delays after symptom onset, leading to irreversible organ damage by the time treatment is initiated. [4] Historically, enzyme replacement therapy (ERT) with agalsidase alfa or beta has been the mainstay of disease-specific treatment, demonstrating benefits in substrate clearance, renal stabilisation, and reduction in cardiac mass. [5], [6], [7] However, limitations such as intravenous administration, immunogenicity, variable tissue penetration, and the need for lifelong infusions have prompted the development of alternative approaches. [8] Second-generation ERT (e.g. pegunigalsidase alfa) may reduce immunogenicity, [9] but still requires intravenous delivery. In contrast, oral chaperone therapy offers greater convenience, with an expanding evidence base for safety and effectiveness. [10], [11]

Migalastat is a first-in-class oral pharmacological chaperone that selectively binds and stabilises amenable mutant forms of α-Gal, facilitating lysosomal trafficking and restoring enzymatic activity. [12] Approximately 35–50% of individuals with FD carry amenable variants. [13] Clinical trials, including ATTRACT and FACETS, demonstrated that migalastat is non-inferior to ERT in stabilising renal function, with additional benefits such as reduced left ventricular mass and a favourable safety profile. [14], [15] Subsequent long-term and real-world studies have reinforced its multisystem efficacy and tolerability. [10], [11] In the United Kingdom (UK), migalastat was licensed by the Medicines and Health Regulation Authority (MHRA) in 2016 and recommended by the National Institute for Health and Care Excellence (NICE) in 2017 for patients aged ≥16 years with amenable *GLA* variants, provided they meet ERT eligibility. [16]

Despite demonstrated efficacy in clinical trials, long-term real-world data remain necessary, particularly in mixed groups of treatment-naïve and ERT-switch patients. Moreover, existing studies have included limited genotypic data and predominantly short-term outcomes. [10], [11] The present study provides long-term, genotype-informed data from a mixed cohort of adults including those with classical and late-onset *GLA* variants, thereby extending understanding of migalastat effectiveness and tolerability across a broader FD population.

This study aimed to evaluate the real-world effectiveness and tolerability of migalastat in adults with FD, with subgroup comparisons between treatment-naïve (TN) patients and those switched from ERT (TS).

## 2 Methods

### 2.1 Study design and population

Institutional approval was obtained (S19MET08-S), and the study was approved by the Health Research Authority (ID: 262706, REF: 19/HRA/5221). Migalastat was initiated according to amenability information and clinical guidance available at the time of treatment onset. Formal *in-vitro* amenability testing and the Galafold® amenability table (Galafold® (migalastat), Amicus Therapeutics, Inc., Cranbury, NJ, USA) were first introduced around the time of migalastat's regulatory approval (2016 in the UK), and the table has undergone several revisions since then (latest edition, 2024). Some patients in this cohort commenced migalastat as part of early access or clinical trial pathways, and others were started on treatment before the current amenability classifications were established. In a small number of cases, particularly where historical amenability data were limited or the variant was of uncertain significance (VUS), clinical decision-making was supported by the overall phenotype, biochemical profile, and multidisciplinary specialist judgement. VUS were included when supported by biochemical, enzymatic, or clinical evidence of FD. Eligible participants were required to have at least one documented pre-treatment assessment and one follow-up visit ≥12 months after migalastat initiation. Exclusion included incomplete baseline data.

Data were obtained from a previously described longitudinal FD cohort [17] and analysis of electronic health records (EHR) as part of routine clinical care at the Adult Inherited Metabolic Diseases service, Salford Care Organisation, Northern Care Alliance (NCA) National Health Service (NHS) Foundation Trust. At the time of data extraction, five patients had discontinued migalastat (further analysed in results section); no additional patients were lost to follow-up. The study period spanned January 2016 to December 2024, providing up to 8 years of combined migalastat and ERT exposure across the cohort.

### 2.2 Data collection

Baseline data were defined as values recorded prior to initiation of disease specific therapy (either ERT or migalastat) and the most recent measurements at last review (≥ 12 months after migalastat initiation). Variables included demographics, past medical history, concomitant medication, and clinical parameters (heart rate, blood pressure (BP), body mass index (BMI)). Genotype, plasma globotriaosylsphingosine (lyso-Gb3), α-Gal, and renal function were recorded. Renal function was assessed using serum creatinine and estimated glomerular filtration rate (eGFR), calculated with the Modification of Diet in Renal Disease (MDRD) equation for results obtained before July 2021 and the Chronic Kidney Disease Epidemiology Collaboration (CKD-EPI) equation, thereafter, reflecting the standard laboratory transition during the study period. To allow consistent comparison across estimating methods, renal function was analysed categorically using established thresholds of eGFR >90 mL/min/1.73 m^2^ (normal function) and > 60 mL/min/1.73 m^2^ (clinically relevant boundary for CKD). This categorical approach was selected to avoid over-interpretation of minor absolute fluctuations that may reflect age-related decline, intra-individual variability, or methodological differences between estimating equations rather than Fabry-related progression. Urinary albumin-to-creatinine (uACR) and protein-to-creatinine ratios (uPCR) were also collected. Cardiac assessment included electrocardiographic parameters (PR and QTc intervals) and echocardiographic measures: interventricular septal thickness at end-diastole (IVSd), left-ventricular mass (LVM), and left-ventricular mass index (LVMI).

### 2.3 Study endpoints

The primary clinical endpoints were changes in renal and cardiac parameters during migalastat therapy, including eGFR, uPCR, uACR, PR and QTc intervals, IVSd, LVM, and LVMI. Incidence of non-fatal cardiovascular events (NFCVEs), end stage kidney disease (ESKD) requiring renal replacement therapy (RRT) and all-cause mortality were also evaluated as part of the primary clinical assessment. Secondary endpoints included biochemical response, measured by changes in plasma lyso-Gb3 and α-Gal activity from baseline to follow-up. Safety endpoints comprised adverse events leading to discontinuation, organ function decline, or death. NFCVEs were defined as myocardial infarction, acute coronary syndrome, heart failure, clinically significant arrhythmias, transient ischaemic attack, or stroke.

### 2.4 Statistical analysis

Continuous variables are presented as medians with interquartile ranges (IQR), and categorical variables as counts and percentages. Paired within-patient comparisons between baseline and follow-up were performed using the Wilcoxon signed-rank test, as data were not normally distributed. Between-group comparisons used the Mann–Whitney *U* test for continuous variables and chi-squared (χ^2^) or McNemar's tests for categorical variables. Incidence rates of non-fatal cardiovascular events (NFCVEs) were calculated per 1000 person-years of follow-up. A two-sided *p*-value <0.05 was considered statistically significant. All analyses were conducted using SPSS v26 (IBM Corp., Armonk, NY, USA) under institutional licence (University of Manchester).

In addition to primary analyses, prespecified subgroup and sensitivity analyses were performed, including exclusion of individuals carrying variants now classified as non-amenable or untested in the 2024 UK Galafold® amenability table, to assess the robustness of findings.

## 3 Results

### 3.1 Study population and baseline characteristics

**Fig. 1:**
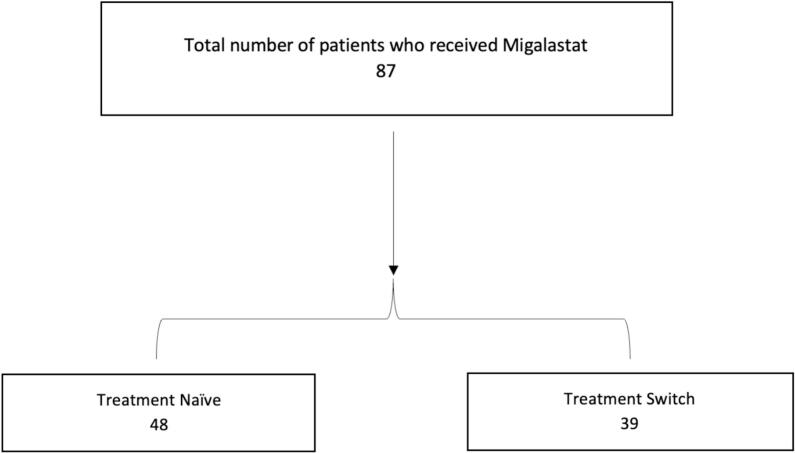
Distribution of patients who received Migalastat by treatment history.

Eighty-seven patients with genetically confirmed FD receiving migalastat were included in the analysis (55 males, 32 females) (Fig. 1). Participant ethnic group was collected via self-report, with all individuals identifying as White British. Baseline characteristics are summarised in Table 1, Table 2 (*Table 1,* *Supplementary Material*). The median age at baseline was 51 years (42–60), and all participants were of white ethnicity. The cohort was predominantly male (63%). Genotypes were classified according to previously published genomic cohorts. [18] Based on this framework, 66.7% of patients carried late-onset variants, 13.8% had classical pathogenic variants and 19.5% were of unknown classification (i.e. VUS). The c.644 A > G (p.Asn215Ser)/ p.N215S variant was the most frequently observed mutation, present in approximately half of all TN patients, reflecting the prevalence of this late-onset form within contemporary FD populations.

**Table 1:** Baseline characteristics of patients who received migalastat, stratified by sex: This table presents the baseline demographic, clinical, and biochemical characteristics of 87 patients who received migalastat, stratified by sex (55 males and 32 females).

Variable	Total (87)	Male (55)	Female (32)	p-Value
Age, years	51 (42–60)	48 (41–58)	56 (45–62)	**0.038**
Heart rate, per min	66 (58–73)	63 (56–71)	68 (63–74)	**0.022**
Systolic BP, mm Hg	133 (121–148)	134 (121–148)	131 (120–148)	0.846
Diastolic BP, mm Hg	80 (74–87)	80 (75–87)	81 (74–88)	0.902
Body mass index	27 (25–31)	27 (25–31)	27 (25–31)	0.900
Smoking	5 (5.7)	4 (7.3)	1 (3.1)	0.423
Hypertension	20 (23)	12 (21.8)	8 (25)	0.734
Diabetes mellitus	7 (8)	5 (9.1)	2 (6.3)	0.639
Hypercholesterolemia	13 (14.9)	6 (10.9)	7 (21.9)	0.167
Transient ischemic attack/ Cerebrovascular accident	5 (5.7)	3 (5.5)	2 (6.3)	0.878
Ischemic heart disease	3 (3.4)	2 (3.6)	1 (3.1)	0.900
Atrial fibrillation	6 (6.9)	4 (7.3)	2 (6.3)	0.856
Asthma	8 (9.2)	5 (9.1)	3 (9.4)	0.965
Enzyme replacement therapy to chaperone switch	39 (44.8)	28 (50.9)	11 (34.4)	0.135

**Genetic Groups** Classical Late-onset Unknown	12 (13.8) 58 (66.7) 17 (19.5)	5 (9.1) 42 (76.4) 8 (14.5)	7 (21.9) 16 (50) 9 (28.1)	**0.040**

**Biochemical variables**				
Creatinine, umol/L	77 (65–90)	84 (76–95)	60 (56–71)	**<0.001**
eGFR, ml/min/1.73m^2^	99 (85–108)	93 (85–107)	100 (84–108)	**<0.001**
uPCR, mg/mmol	10.5 (7–20.7)	10 (6–15)	15 (8.3–28)	**0.009**
uACR, mg/mmol	2.3 (1–12.4)	2.4 (0.63–6.6)	2.2 (1.3–23)	0.262
Lysosomal Gb3, ng/ml (baseline)	5.3 (3.7–8.9)	5.6 (3.8–9.2)	4.9 (2.9–8)	0.056
Alpha-galactosidase A enzyme level, nmol/ml/h (baseline)(*n* = 66)	0.6 (0.25–1.3)	0.4 (0.2–0.6)	2.7 (1.4–3.7)	**<0.001**

Significant sex-based differences were observed in age, heart rate, genetic group distribution, and several biochemical markers including creatinine, eGFR, uPCR, and α-Gal levels which is expected given the X-linked nature of FD.

BP – blood pressure; BMI – body mass index; TIA/CVA – transient ischaemic attack / cerebrovascular accident; IHD – ischaemic heart disease; AF – atrial fibrillation; ERT – enzyme replacement therapy; eGFR – estimated glomerular filtration rate; uPCR – urine protein-to-creatinine ratio; uACR – urine albumin-to-creatinine ratio; GB3 – globotriaosylceramide; nmol/ml/h – nanomoles per millilitre per hour; IQR – interquartile range.

**Table 2:** Baseline characteristics of treatment-naïve (TN) and treatment-switch (TS) patients receiving migalastat.

Variable	Treatment naïve (48)	Treatment switch (39)	p-Value
Age, years	57 (44–63)	47 (40–54)	**0.002**
Sex, male	27 (56.3)	28 (71.8)	0.135
Heart rate, per min	68 (61–74)	62 (55–70)	**0.012**
Systolic BP, mm Hg	131 (119–148)	135 (127–145)	0.762
Diastolic BP, mm Hg	80 (75–90)	80 (74–86)	0.478
Body mass index	27 (25–31)	27 (25–30)	0.845
Smoking	2 (4.2)	3 (7.7)	0.456
Hypertension	14 (29.2)	6 (15.4)	0.129
Diabetes mellitus	6 (12.5)	1 (2.6)	0.090
Hypercholesterolemia	7 (14.6)	6 (15.4)	0.917
Transient ischemic attack/ Cerebrovascular accident	3 (6.3)	2 (5.1)	0.823
Ischemic heart disease	2 (4.2)	1 (2.6)	0.684
Atrial fibrillation	5 (10.4)	1 (2.6)	0.151
Asthma	6 (12.5)	2 (5.1)	0.237

**Genetic Groups** Classical Late-onset Unknown p.N215S	5 (10.4) 34 (70.8) 9 (18.8) 25 (52.1)	7 (17.9) 24 (61.5) 8 (20.5) 19 (48.7)	0.549

**Biochemical variables**			
Creatinine, umol/L	74 (64–90)	77 (67–88)	0.778
eGFR, ml/min/1.73m^2^	92 (78–106)	103 (89–109)	0.026
uPCR, mg/mmol	12 (7–21)	9 (7–16)	**0.049**
uACR, mg/mmol	3.7 (1.3–13.5)	1.8 (1–4.3)	0.159
Lysosomal Gb3, ng/ml (baseline)	5.3 (3.8–8.1)	5.3 (3.5–9.9)	0.187
Alpha-galactosidase A enzyme level, nmol/ml/h (baseline) (n = 66)	0.5 (0.2–1.6)	0.6 (0.3–1)	0.499

This table summarises baseline demographic, clinical, and biochemical features of 87 patients, stratified by treatment status (TN, n = 48; TS, n = 39). TN patients were significantly older, reflecting enrichment of the cohort with the late onset.

Significant sex-based differences were evident across demographic and biochemical parameters. Males demonstrated higher serum creatinine and uPCR, and lower eGFR and α-Gal activity, compared with females (all *p* < 0.05). There was no difference in cardiovascular disease burden at baseline between males and females, or between the TN and TS groups. Males also had a lower resting heart rate (63 vs 68 beats per minute, *p* = 0.022). These patterns are consistent with the recognised sex-related differences in FD. TN patients were significantly older (median 57 vs 47 years; *p* = 0.002) and more frequently carried the late-onset c.644 A > G (p.Asn215Ser)/p.N215S variant (52.1% vs 48.7%), whereas TS patients exhibited higher baseline eGFR (*p* = 0.026) and lower uPCR (*p* = 0.049), consistent with earlier diagnosis and prior exposure to ERT. The median follow-up duration differed substantially between groups; 3.6 years (2.4–4.5) for TN patients and 11.8 years (6.0–14.9) for TS patients, providing an extended observation period for long-term outcomes among those previously treated with ERT. Among TS patients, median exposure to ERT prior to transition was 4.7 years (2.7–9.6), after which patients received migalastat for 3.6 years (3.2–4.1).

### 3.2 Safety and tolerability

Across the study cohort, migalastat treatment was generally well tolerated, though a small number of patients experienced adverse clinical outcomes during follow-up (*Table 2, Supplementary Material*). Among 87 participants, six males (6.9%) reported clinically significant events, comprising four TN and two TS patients. Five discontinued migalastat (two cardiac progression, one renal decline, one gastrointestinal intolerance, one rising lyso-Gb3), and one Fabry-related death occurred in the TN group.

Documented reasons included progressive cardiac involvement in two cases, declining renal function (eGFR <30 mL/min/1.73 m^2^) in one (with reported good compliance in those with cardiac and renal worsening), and gastrointestinal intolerance in one patient. A further TS patient discontinued following a sustained rise in plasma lyso-Gb3, despite otherwise stable clinical parameters. All five patients reverted to ERT and had clinical stabilisation, and improved tolerance. No episodes of anaphylaxis, hepatic toxicity, or drug-induced arrhythmia were reported, and there were no treatment interruptions due to laboratory abnormalities. Overall, migalastat was tolerated in most patients during extended follow-up, and adverse events were generally consistent with the reported disease course.

### 3.3 Primary endpoints

#### 3.3.1 Renal function

Renal parameters were assessed using serum creatinine, eGFR, and measures of proteinuria (Table 3). At baseline, 50% of TN patients and 84.6% of TS patients had an eGFR >90 mL/min/1.73 m^2^. These proportions declined to 29% and 30.8% at follow-up (*p* = 0.037 for TN; *p* < 0.001 for TS). Despite this shift, most patients maintained an eGFR >60 mL/min/1.73 m^2^ at follow-up (70.8% TN; 89.7% TS), and no patient progressed to end-stage kidney disease. Proteinuria remained stable, with no significant changes in uPCR or uACR in either group. Blood pressure also remained unchanged across time points. Overall, renal function remained within clinically relevant thresholds, and although the proportion of patients with eGFR >90 mL/min/1.73 m^2^ declined, the majority preserved eGFR >60 mL/min/1.73 m^2^ and stable proteinuria throughout follow-up.

**Table 3:** Renal and cardiovascular parameters at baseline and follow-up in treatment-naïve and treatment-switch patients receiving migalastat.

	Treatment naïve (*N* = 48)			Treatment switch (*N* = 39)		
Variables	Baseline	Follow-up	p-Value	Baseline	Follow-up	p-Value
**Biochemical**						
eGFR >90, ml/min/1.73m^2^	24/48 (50%)	14/48 (29%)	**0.037**	33/39 (84.6%)	12/39 (30.8%)	**<0.001**
eGFR >60, ml/min/1.73m^2^	40/48 (83.3%)	34/48 (70.8%)	0.145	39/39 (100%)	35/39 (89.7%)	0.157
uPCR, mg/mmol	12 (7–21)	13 (8–30)	1.000	9 (7–16)	11 (5–19)	0.175
uACR, mg/mmol	3.7 (1.3–13.5)	4.55 (1.29–12.4)	0.343	1.8 (1–4.3)	2.15 (0.6–16)	0.564

**Cardiovascular**						
PR interval (ms)	158 (146–176)	169 (146–192)	0.067	156 (133–176)	163 (142.5–180.5)	0.063
QTc interval (ms)	435 (408–454.5)	444 (411–466.5)	0.319	418 (397.5–434)	442.5 (422–469)	**<0.001**
Systolic BP, mm Hg	133 (120–148)	132 (119–152)	0.257	133 (126–145)	129 (119–140)	0.060
Diastolic BP, mm Hg	80 (74–89)	82 (71–90)	0.301	80 (74–87)	82 (76–90)	0.340
Intraventricular septum at end-diastole (IVSd) (cm)	1.3 (1–1.7)	1.3 (1.2–1.65)	0.308	1.3 (0.96–1.6)	1.4 (1.2–1.7)	0.214
LVM (g)	224.6 (176.6–328.6)	237.1 (173.45–299.4)	0.739	233.5 (175.8–303.4)	219.1 (176.5–257.6)	0.110
LVMI (g/m^2^)	110.9 (93.1–154.6)	113.8 (93–161.8)	0.736	121.7 (96.1–138.7)	105.3 (90.8–136.3)	0.088

Values are presented as n (%) or median (interquartile range). Comparisons between baseline and follow-up were performed using the Wilcoxon signed-rank test for continuous variables and McNemar's test for categorical variables. A *p*-value <0.05 was considered statistically significant. Renal function remained largely preserved in both groups, with stable proportions of patients maintaining eGFR >60 mL/min/1.73 m^2^ and no progression to ESKD. Proteinuria measures (uPCR and uACR) showed no significant change. Blood pressure remained stable across groups. In treatment-naïve patients (TN), cardiac electrical and structural parameters were unchanged. In the treatment-switch group, QTc interval increased significantly, although values generally remained within normal or mildly abnormal ranges.

eGFR – estimated glomerular filtration rate; ESKD, end-stage kidney disease; IQR - interquartile range; IVSd - interventricular septal thickness at end-diastole; LVM - left ventricular mass; LVMI - left ventricular mass index; ms - milliseconds; QTc - corrected QT interval; uACR - urine albumin-to-creatinine ratio; uPCR - urine protein-to-creatinine ratio.

#### 3.3.2 Cardiovascular parameters

Cardiac structural and conduction parameters are summarised in Table 3. In the TN group, PR interval, QTc interval, IVSd, LVM, and LVMI showed no significant changes over the follow-up period (all *p* > 0.05). In the TS group, QTc increased significantly (418 to 442.5 ms, *p* **<** **0.001**), although values remained within normal or only mildly prolonged limits (QTc <460 ms in females, <450 ms in males). No significant changes were observed in PR interval, IVSd, LVM, LVMI, or blood pressure. Overall, cardiac structure and conduction measurements remained broadly stable in both treatment groups. The isolated increase in QTc among TS patients likely reflects a combination of underlying disease progression, physiological variability, and longer follow-up duration rather than a migalastat-specific effect.

#### 3.3.3 Left ventricular mass and geometry (sex-stratified analysis)

Sex-stratified analysis demonstrated the expected higher LVMI values in males at baseline. Among TN patients, median baseline LVMI was 141.7 g/m^2^ (107.9–213.1) in males and 96.2 g/m^2^ (79.2–136.9) in females, compared with 111.6 g/m^2^ (98.7–140.0) and 105.3 g/m^2^ (89.5–132.8), respectively, in the TS group. Follow-up LVMI values declined modestly in all subgroups, with median ΔLVMI values of −6.3 g/m^2^ (*p* = 0.126) in TN males, −3.9 g/m^2^ (*p* = 0.317) in TN females, −11.1 g/m^2^ (*p* = 0.055) in TS males, and − 10.4 g/m^2^ (*p* = 0.146) in TS females. None of these changes reached statistical significance, and there were no sex differences in ΔLVMI within either treatment group (TN *p* = 0.505; TS *p* = 0.880).

Left ventricular hypertrophy (LVH), defined as LVMI >115 g/m^2^ in males or > 95 g/m^2^ in females, decreased modestly across all subgroups. In TN males, LVH declined from 73.1% to 61.5% (*p* = 0.39) and in TN females from 46.7% to 40.0% (*p* = 0.71). In the TS cohort, LVH declined from 45.5% to 36.4% in males (*p* = 0.58) and from 38.1% to 28.6% in females (*p* = 0.55). None of these changes were statistically significant.

#### 3.3.4 Clinical events

**Fig. 2:**
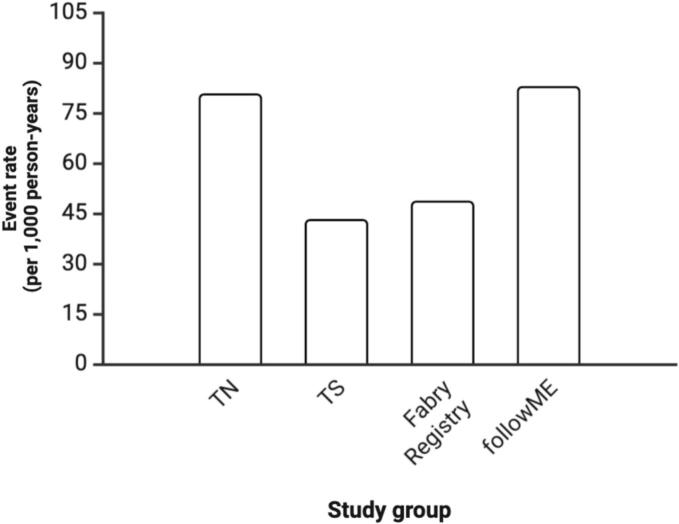
Event rates in current and historical Fabry disease cohorts: This bar chart compares NFCVE rates across TN and TS groups in the current study with those reported in the *Fabry Registry*[19] and the *followME* study [11]. Event rates are shown per 1000 person-years. The *Fabry Registry* reported a range of 40–58 events, and the midpoint (49) is displayed here for visual comparison. Likewise, *followME* reported cardiac events of 83.2 per 1000 person-years displayed here. Differences in follow-up duration, event definitions, and treatment exposure between studies should be considered when interpreting the data.

During follow-up, non-fatal cardiovascular events (NFCVEs) occurred in 14/48 TN (29.2%) and 20/39 TS (51.3%) patients (*p* = 0.060) (Table 4). No patients progressed to end-stage kidney disease requiring renal replacement therapy, and one Fabry-related death occurred in the TN group. Follow-up duration differed substantially between groups (median 3.6 years in TN vs 11.8 years in TS), influencing cumulative event counts. When adjusted for exposure time, the incidence of NFCVEs was 81 per 1000 person-years in TN patients and 43.5 per 1000 person-years in TS patients (Fig. 2).

**Table 4:** Clinical outcomes and follow-up duration in treatment-naïve (TN) vs treatment-switch (TS) patients receiving migalastat

Outcome	Treatment naïve (48)	Treatment switch (39)	p-Value
NFCVE	14 (29.2%)	20 (51.3%)	0.060
All-cause mortality	1(2.6%)	0	0.264
Follow-up, years	3.6 (2.4–4.5)	11.8 (6.02–14.9)	**<0.001**

This table presents the incidence of new non-fatal cardiovascular events (NFCVEs), all-cause mortality, and duration of follow-up in TN and TS patients. NFCVE was defined as the first occurrence of a Fabry-related cardiovascular complication following migalastat initiation, including documented arrhythmia, heart failure,

### 3.4 Secondary endpoints

#### 3.4.1 Biochemical outcomes

**Fig. 3:**
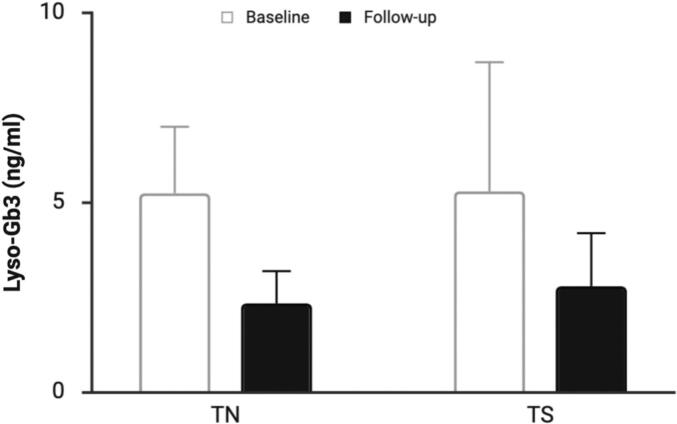
Change in plasma lyso-Gb3 concentration in treatment-naïve (TN) and treatment-switch (TS) patients receiving migalastat. Bars represent median values with interquartile ranges (IQR). In the TN group, lyso-Gb3 declined from 5.3 (3.8–8.1) to 2.4 (1.3–3.5) ng/ml, corresponding to a 55.2% reduction. In the TS group, lyso-Gb3 decreased from 5.3 (3.5–9.9) to 2.8 (1.4–5.8) ng/ml, representing a 47.2% reduction. Both groups demonstrated biochemical response to therapy.

Plasma lyso-Gb3 concentrations were available at baseline and follow-up for 84 patients (Fig. 3). Levels declined in both TN and TS groups. In TN patients, median lyso-Gb3 decreased from 5.3 (3.8–8.1) to 2.4 (1.4–3.5) ng/mL, representing a 55.2% (48.5–60.6) reduction, while in TS patient's levels fell from 5.3 (3.5–9.9) to 2.8 (1.4–5.8) ng/mL (47.2% (43.2–56.6) reduction). The assay reference range was <1.8 ng/mL, and biochemical normalisation occurred in 35.4% (17/48) of TN and 30.8% (12/39) of TS patients. Follow-up lyso-Gb3 data were missing for three individuals, and only one patient showed no measurable reduction. Enzyme α-Gal activity was assessed in a smaller subset of 26 patients. Enzymatic activity showed a tendency to increase during migalastat therapy, but interpretation is limited given the small sample size and the timing of sampling relative to dosing, which can influence measured activity.

### 3.5 Further subgroup analysis

#### 3.5.1 Genotype distribution and outcome

In the TN group, c.644 A > G (p.Asn215Ser)/p.N215S accounted for 52.1% of patients. Recurrent non-N215S variants included c.902G > A (p.Arg301Gln)/p.R301Q (10.4%) and c.695 T > C (p.Ile232Thr)/p.I232T (10.4%), with smaller numbers of c.613C > A (p.Pro205Thr)/p.P205T (4.2%) and c.641C > T (p.Pro214Leu)/p.P214L (4.2%), and isolated cases of c.154 T > G (p.Cys52Gly)/p.C52G, c.955 A > T (p.Ile319Phe)/p.I319F, and c.1066C > T (p.Arg356Trp)/p.R356W.

The TS cohort demonstrated a similar predominance of c.644 A > G (p.Asn215Ser)/p.N215S (59.0%), but with greater allelic diversity, including c.776C > G (p.Pro259Arg)/p.P259R (10.3%), c.902G > A (p.Arg301Gln)/p.R301Q (7.7%), and rarer late-onset variants such as c.1202C > T(p.Thr401Ile)/p.T401I,c.613C > A(p.Pro205Thr)/p.P205T,c.831G > C(p.Trp277Cys)/p.W277C, c.1024C > T (p.Arg342Ter)/p.R342X, c.769G > C (p.Ala257Pro)/p.A257P, c.724 A > T (p.Ile242Phe)/p.I242F, c.713G > A (p.Ser238Asn)/p.S238N, and c.19G > T (p.Glu7Ter)/p.E7X. This pattern reflects the enrichment of late-onset variants in both treatment groups, with broader genetic heterogeneity in the TS cohort consistent with longer disease duration and prior therapy exposure.

NFCVEs occurred across multiple genotypes, including c.644 A > G (p.Asn215Ser)/p.N215S, c.902G > A(p.Arg301Gln)/p.R301Q,c.1024C > T(p.Arg342Ter)/p.R342X,c.955 A > T (p.Ile319Phe)/p.I319F,c.613C > A (p.Pro205Thr)/p.P205T, c.776C > G (p.Pro259Arg)/p.P259R, c.769G > C(p.Ala257Pro)/p.A257P,c.713G > A(p.Ser238Asn)/p.S238N,c.19G > T (p.Glu7Ter)/p.E7X, and c.1202C > T (p.Thr401Ile)/p.T401I.

The predominance of p.N215S among these events reflects its frequency within the cohort rather than a mutation-specific risk. Treatment discontinuations or death occurred in individuals carrying c.644 A > G(p.Asn215Ser)/p.N215S,c.902G > A(p.Arg301Gln)/p.R301Q,c.695 T > C(p.Ile232Thr)/p.I232T, c.1066C > T (p.Arg356Trp)/p.R356W, and c.1235C > A (p.Thr412Asn)/p.T412N. When stratified by genotype class (classical, late-onset, and unclassified), no clear clustering of NFCVEs was observed. Among those associated with adverse outcomes, p.N215S, p.R301Q, and p.I232T are considered late-onset forms, whereas p.R356W and p.T412N are associated with classical phenotypes.

#### 3.5.2 Patients with non-amenable variants (based on 2024 UK Galafold® table)

According to the updated 2024 UK Galafold® amenability table, four patients carried GLA variants now classified as non-amenable (p.C52G, p.R342X, p.E7X) or not tested (p.T401I). One of these individuals belonged to the TN group (p.C52G) and three to the TS group (p.R342X, p.E7X, p.T401I). All four commenced migalastat before contemporary amenability classifications were available, during a period when treatment decisions were guided by earlier in-vitro data, clinical phenotype, and specialist judgement. Their inclusion therefore reflects real-world prescribing practice at a time when amenability criteria were still evolving.

Exclusion of these four patients did not materially alter renal, biochemical, or cardiovascular results (*Table 3, Supplementary Material*). The decline in the proportion of patients with eGFR >90 mL/min/1.73 m^2^ persisted in both TN and TS groups, and most patients continued to maintain eGFR >60 mL/min/1.73 m^2^. Measures of proteinuria (uPCR, uACR) and blood pressure remained stable. Cardiac conduction and structural parameters were unchanged in the TN group, while QTc prolongation remained statistically significant in the TS group. The reduction in LVMI in TS patients became more pronounced and reached statistical significance in the sensitivity analysis, whereas IVSd and LVM continued to demonstrate no significant change. In addition, After excluding the four patients, the distribution of NFCVEs was unchanged, with events occurring in 14/47 TN (29.8%) and 17/36 TS (47.2%) patients; this difference remained non-significant (*p* = 0.162).

#### 3.5.3 c.644 A > G (p.Asn215Ser)/p.N215S variant

Among TN patients, 25 individuals (52.1%) carried the p.N215S variant. Cardiac and conduction parameters in this subgroup remained stable over the observation period (*Table 4, Supplementary Material*). No statistically significant changes were observed in PR or QTc intervals, IVSd, LVM, or LVMI (all *p* > 0.05). Median LVM and LVMI values remained within reference limits for age and sex throughout follow-up. Cardiac structure and conduction were maintained in patients with the p.N215S genotype during early migalastat therapy. However, the small sample size and relatively short follow-up period for this subgroup limit definitive conclusions regarding long-term progression.

## 4 Discussion

This single-centre, real-world study provides additional evidence on migalastat use in adults with FD and amenable *GLA* variants. Our findings are broadly consistent with prior trials and registry data. [14], [15] Recent evidence has highlighted the substantial renal burden in FD, with >60% lifetime CKD risk even at near-normal indices. [20] In our cohort, renal function on migalastat appeared largely preserved, with most patients maintaining eGFR >60 mL/min/1.73 m^2^. The decline in the proportion with eGFR >90 mL/min/1.73 m^2^ is notable but is plausibly explained by expected age-related loss (around 1 mL/min/1.73 m^2^ per year) and the laboratory transition to a newer eGFR estimating equation during the study period, rather than accelerated Fabry-related decline. Proteinuria indices also remained stable, consistent with prior real-world series. [10], [11], [21] However, data from *Pisani* et al. [22] indicate that eGFR decline accelerated after switching from agalsidase beta to migalastat (−1.96 vs −0.85 mL/min/1.73 m^2^ per year), with proteinuria increases most evident in classical patients, underscoring phenotype-specific variability. In contrast, the FAMOUS prospective studies [23], [24] reported steeper short-term eGFR declines under migalastat (−6.9 in females and − 5.0 mL/min/1.73 m^2^ in males at 12 months), with attenuation after two years. Importantly, the authors noted that renal loss was most pronounced in patients with systolic BP < 120 mmHg, suggesting that low BP may confound renal outcomes. [23] In our cohort, blood pressure remained stable, and most patients maintained eGFR >60 mL/min/1.73 m^2^ with no significant proteinuria, supporting the view that renal trajectories under migalastat are influenced by baseline phenotype, BP status, and methodological factors as well as treatment response.

Cardiac outcomes in our cohort remained largely stable over time with migalastat therapy. Despite the high prevalence of p.N215S, the incidence of NFCVEs and the overall pattern of echocardiographic findings were comparable to those reported in other real-world Fabry cohorts [11], [19]. Unlike ATTRACT [14], our primary analysis did not demonstrate significant reductions in LVMI, likely reflecting baseline characteristics, including the predominance of normal or only mildly increased LV wall thickness. In the TS group, QTc increased modestly, although values remained within or only slightly above accepted reference limits. IVSd and LVM remained unchanged. Notably, when individuals with non-amenable or untested variants were excluded, LVMI reduction in the TS cohort became statistically significant, suggesting a treatment-associated effect that may be detectable only in fully amenable genotypes. Interpretation of TS trajectories is complicated by the unknown durability of prior ERT effects. These findings contrast with the deterioration in LV parameters reported by *Pisani* et al. [22] among classical patients switched from agalsidase beta to migalastat, but align more closely with FAMOUS [23], [24], in which LVMI reduction was most evident in patients with baseline hypertrophy. In our cohort, the prevalence of LV hypertrophy declined modestly across subgroups without statistical significance, suggesting maintenance or slight regression rather than progressive remodelling, although minor inter-operator variation in echocardiographic measurement may have contributed. Collectively, the stability observed likely reflects attenuation of disease progression in late-onset variants, although a slower inherent disease trajectory cannot be excluded. Consistent with MAIORA [25], TN patients maintained preserved cardiac structure and function, and we provide, to our knowledge, the first evidence of PR and QTc interval stabilisation in migalastat-treated TN patients.

A comparison with previously published Fabry cohorts demonstrated broadly similar clinical event frequencies (Fig. 2). The *Fabry Registry* [19] (predominantly ERT-treated) reported approximately 40–58 events per 1000 person-years, and the *followME* [11] study (migalastat-treated) reported 83.2 cardiac events per 1000 person-years. However, direct comparison should be interpreted cautiously, as these cohorts differed in event definitions, baseline risk profiles, and follow-up duration. Overall, the data indicate that cardiovascular event rates in this cohort were consistent with prior real-world observations but confirm that ongoing cardiovascular surveillance remains essential during long-term migalastat therapy.

Migalastat was not universally effective in our cohort, with a minority of patients experiencing disease progression or intolerance necessitating discontinuation. One patient with a classical mutation reverted to ERT, while additional cases of cardiac progression occurred in individuals with late-onset variants, including one early-onset presentation associated with a VUS. Our findings align with previous reports describing subsets of patients with renal decline and variable cardiac outcomes, as well as registry data indicating worsening renal and cardiac parameters in classical patients post-switch, in contrast to greater stability among those with late-onset variants. [22], [23], [24] In contrast, long-term trial follow-up in females has demonstrated preservation of renal function, stable cardiac indices, and a low incidence of major clinical events during migalastat therapy, with benefits observed irrespective of baseline disease severity, including LVH, multiorgan involvement, or prior events. [26] These observations reinforce that mutation amenability alone may not guarantee clinical benefit; genotype, sex, baseline organ involvement, and age at treatment initiation remain key determinants of therapeutic response. In our cohort, adverse outcomes occurred across both classical and late-onset genotypes, suggesting that event occurrence may reflect individual disease burden and baseline organ involvement rather than genotype-specific susceptibility.

Biochemically, both TN and TS groups demonstrated reductions in lyso-Gb3, consistent with previous reports [10], [11], and in the sensitivity analysis the reduction in LVMI in TS patients became statistically significant. Interpretation of changes in α-Gal activity remains limited by assay and pharmacokinetic variability, with peak values at 3–6 h and troughs at 24–72 h post-dose [[27], [28], [29]]. Standardised sampling protocols are required before α-Gal activity can be considered a reliable biomarker [30]. In TS patients, baseline measurements reflected values obtained prior to any disease-specific therapy rather than immediately before switching, and the subsequent trajectories therefore represent true stabilisation rather than a blunted response influenced by preceding ERT. The durability of prior ERT effects on myocardial mass is unknown and may persist for years, making it challenging to fully isolate the incremental effect of migalastat in switched patients. FACETS [15] did not meet its primary histological endpoint, partly because some participants were later found to harbour non-amenable variants. In our cohort, the inclusion of patients with variants initially reported as VUS or later reclassified as non-amenable represented a small proportion of the overall population and reflected historical prescribing practice during periods when amenability designations were less well defined. The methodology underlying amenability testing has evolved substantially over time, from early in-vitro overexpression systems to the current Good Laboratory Practice (GLP)–validated HEK (human embryonic kidney) cell assay that underpin contemporary Galafold® amenability classifications. [27], [30], [31] As a result, some variants have been reclassified as new functional data have emerged. Importantly, exclusion of these individuals in sensitivity analyses did not change the principal renal or most cardiac findings, supporting the robustness of our results. Lyso-Gb3 was the only routinely available biomarker; most patients demonstrated reductions, though few achieved complete normalisation, and one showed a paradoxical increase despite documented adherence. At present, no evidence defines the degree of lyso-Gb3 change required to confer long-term clinical benefit, and stable or rising levels remain difficult to interpret. These uncertainties underscore the need for consensus biochemical monitoring strategies and the development of improved biomarkers that more closely reflect disease activity. While the oral route of migalastat offers practical advantages, accurate amenability classification and longitudinal biochemical surveillance remain essential to identify suboptimal responders and optimise treatment decisions.

### 4.1 Limitations and further research

This single-centre observational study has several limitations. The modest sample size, predominance of late-onset variants (particularly p.N215S) and shorter follow-up in TN patients may restrict generalisability to individuals with classical FD. Enzyme activity measurements were not standardised, extra-cardiac manifestations were not systematically assessed, and reliance on single pre- and post-treatment biochemical values may introduce variability and selection bias. Current UK guidance, including NICE, recommends migalastat for adults with amenable variants and preserved renal function (eGFR >30 mL/min/1.73 m^2^) [16], whereas its use is not advised in advanced kidney disease due to limited trial evidence [12], [14], [15], [32], [33], [34]. The evolving nature of amenability classification represents an important consideration when interpreting real-world treatment outcomes. Despite these limitations, this study provides real-world evidence of clinical and biochemical stabilisation in genotypically diverse TN and TS cohorts, underscoring the need for larger, multicentre, genotype-stratified studies with standardised biomarkers to define long-term effectiveness and predictors of response.

## 5 Conclusion

In this single-centre, real-world cohort of adults with FD and amenable, predominantly late-onset, *GLA* variants, migalastat therapy was associated with biochemical improvement and stability of renal and cardiac parameters during long-term follow-up. However, a subset of patients demonstrated disease progression or treatment intolerance, suggesting that response may be influenced by baseline disease burden and phenotype. These findings emphasise that variant amenability, while necessary, may not be sufficient to predict therapeutic benefit; genotype- and phenotype informed treatment selection remains essential to optimise outcomes and advance precision management in FD.

## CRediT authorship contribution statement

**Eamon P. McCarron:** Writing – review & editing, Writing – original draft, Methodology, Investigation, Formal analysis, Data curation, Conceptualization. **Rajkumar Chinnadurai:** Writing – original draft, Methodology, Formal analysis, Data curation. **Karolina M. Stepien:** Writing – review & editing. **John Bassett:** Writing – review & editing. **Jonathan Meyer:** Writing – review & editing. **Reena Sharma:** Writing – review & editing, Validation, Data curation. **Peter Woolfson:** Writing – review & editing, Formal analysis. **Ana Jovanovic:** Supervision, Conceptualization.

## Declaration of competing interest

None.

## Data Availability

Data will be made available on request.

## References

[1] Germain D.P. (2010). Fabry disease. Orphanet J. Rare Dis..

[2] Germain D.P., Arad M., Burlina A., Elliott P.M., Falissard B., Feldt-Rasmussen U., Hilz M.J., Hughes D.A., Ortiz A., Wanner C., Weidemann F., Spada M. (2019). The effect of enzyme replacement therapy on clinical outcomes in female patients with Fabry disease - a systematic literature review by a European panel of experts. Mol. Genet. Metab..

[3] Arends M., Biegstraaten M., Hughes D.A., Mehta A., Elliott P.M., Oder D., Watkinson O.T., Vaz F.M., van Kuilenburg A.B.P., Wanner C., Hollak C.E.M. (2017). Retrospective study of long-term outcomes of enzyme replacement therapy in Fabry disease: analysis of prognostic factors. PLoS One.

[4] Ezgu F., Alpsoy E., Bicik Bahcebasi Z., Kasapcopur O., Palamar M., Onay H., Ozdemir B.H., Topcuoglu M.A., Tufekcioglu O. (2022). Expert opinion on the recognition, diagnosis and management of children and adults with Fabry disease: a multidisciplinary Turkey perspective. Orphanet J. Rare Dis..

[5] Arends M., Biegstraaten M., Wanner C., Sirrs S., Mehta A., Elliott P.M., Oder D., Watkinson O.T., Bichet D.G., Khan A., Iwanochko M., Vaz F.M., van Kuilenburg A.B.P., West M.L., Hughes D.A., Hollak C.E.M. (2018). Agalsidase alfa versus agalsidase beta for the treatment of Fabry disease: an international cohort study. J. Med. Genet..

[6] Eng C.M., Guffon N., Wilcox W.R., Germain D.P., Lee P., Waldek S., Caplan L., Linthorst G.E., Desnick R.J. (2001). International collaborative Fabry disease study group. Safety and efficacy of recombinant human alpha-galactosidase a replacement therapy in Fabry’s disease. N. Engl. J. Med..

[7] Weidemann F., Niemann M., Störk S., Breunig F., Beer M., Sommer C., Herrmann S., Ertl G., Wanner C. (2013). Long-term outcome of enzyme-replacement therapy in advanced Fabry disease: evidence for disease progression towards serious complications. J. Intern. Med..

[8] Umer M., Kalra D.K. (2023). Treatment of Fabry disease: established and emerging therapies. Pharmaceuticals (Basel).

[9] Schiffmann R., Goker-Alpan O., Holida M., Giraldo P., Barisoni L., Colvin R.B., Jennette C.J., Maegawa G., Boyadjiev S.A., Gonzalez D., Nicholls K., Tuffaha A., Atta M.G., Rup B., Charney M.R., Paz A., Szlaifer M., Alon S., Brill-Almon E., Chertkoff R., Hughes D. (2019). Pegunigalsidase alfa, a novel PEGylated enzyme replacement therapy for Fabry disease, provides sustained plasma concentrations and favorable pharmacodynamics: a 1-year phase 1/2 clinical trial. J. Inherit. Metab. Dis..

[10] Hughes D.A., Bichet D.G., Giugliani R., Hopkin R.J., Krusinska E., Nicholls K., Olivotto I., Feldt-Rasmussen U., Sakai N., Skuban N., Sunder-Plassmann G., Torra R., Wilcox W.R. (2023). Long-term multisystemic efficacy of migalastat on Fabry-associated clinical events, including renal, cardiac and cerebrovascular outcomes. J. Med. Genet..

[11] Hughes D.A., Sunder-Plassmann G., Jovanovic A., Brand E., West M.L., Bichet D.G., Pisani A., Nowak A., Torra R., Khan A., Azevedo O., Lehman A., Linhart A., Rutecki J., Giuliano J.D., Krusinska E., Nordbeck P. (2025). Renal and multisystem effectiveness of 3.9 years of migalastat in a global real-world cohort: results from the followME Fabry Pathfinders registry. J. Inherit. Metab. Dis..

[12] McCafferty E.H., Scott L.J. (2019). Migalastat: a review in Fabry disease. Drugs.

[13] Riccio E., Zanfardino M., Ferreri L., Santoro C., Cocozza S., Capuano I., Imbriaco M., Feriozzi S., Pisani A., AFFIINITY Group (2020). Switch from enzyme replacement therapy to oral chaperone migalastat for treating Fabry disease: real-life data. Eur. J. Hum. Genet..

[14] Hughes D.A., Nicholls K., Shankar S.P., Sunder-Plassmann G., Koeller D., Nedd K., Vockley G., Hamazaki T., Lachmann R., Ohashi T., Olivotto I., Sakai N., Deegan P., Dimmock D., Eyskens F., Germain D.P., Goker-Alpan O., Hachulla E., Jovanovic A., Lourenco C.M., Narita I., Thomas M., Wilcox W.R., Bichet D.G., Schiffmann R., Ludington E., Viereck C., Kirk J., Yu J., Johnson F., Boudes P., Benjamin E.R., Lockhart D.J., Barlow C., Skuban N., Castelli J.P., Barth J., Feldt-Rasmussen U. (2017). Oral pharmacological chaperone migalastat compared with enzyme replacement therapy in Fabry disease: 18-month results from the randomised phase III ATTRACT study. J. Med. Genet..

[15] Germain D.P., Hughes D.A., Nicholls K., Bichet D.G., Giugliani R., Wilcox W.R., Feliciani C., Shankar S.P., Ezgu F., Amartino H., Bratkovic D., Feldt-Rasmussen U., Nedd K., Sharaf El Din U., Lourenco C.M., Banikazemi M., Charrow J., Dasouki M., Finegold D., Giraldo P., Goker-Alpan O., Longo N., Scott C.R., Torra R., Tuffaha A., Jovanovic A., Waldek S., Packman S., Ludington E., Viereck C., Kirk J., Yu J., Benjamin E.R., Johnson F., Lockhart D.J., Skuban N., Castelli J., Barth J., Barlow C., Schiffmann R. (2016). Treatment of Fabry’s disease with the pharmacologic chaperone migalastat. N. Engl. J. Med..

[16] National Institute for Health and Care Excellence (NICE) (2017). https://www.nice.org.uk/guidance/hst4/chapter/1-Recommendations.

[17] EP McCarron, Chinnadurai R., Meyer J., Anderson T., Stepien K.M., Sharma R., Woolfson P., Jovanovic A. (2025). Real-world clinical outcomes in adult patients with Fabry disease: a 20-year retrospective observational cohort study from a single centre. Mol. Genet. Metab. Rep..

[18] Sakuraba H., Tsukimura T., Togawa T., Tanaka T., Ohtsuka T., Sato A., Shiga T., Saito S., Ohno K. (2018). Fabry disease in a Japanese population-molecular and biochemical characteristics. Mol. Genet. Metab. Rep..

[19] Ortiz A., Abiose A., Bichet D.G., Cabrera G., Charrow J., Germain D.P., Hopkin R.J., Jovanovic A., Linhart A., Maruti S.S., Mauer M., Oliveira J.P., Patel M.R., Politei J., Waldek S., Wanner C., Yoo H.W., Warnock D.G. (2016). Time to treatment benefit for adult patients with Fabry disease receiving agalsidase β: data from the Fabry registry. J. Med. Genet..

[20] Mannan F., Chinnadurai R., Wiltshire R., Hansel J., Stepien K.M., Sharma R., Wilcox G., McCarron E., Kalra P.A., Jovanovic A. (2025). Epidemiology and early predictors of Fabry nephropathy: evaluation of long-term outcomes from a national Fabry centre. J. Nephrol..

[21] Bichet D.G., Torra R., Wallace E., Hughes D., Giugliani R., Skuban N., Krusinska E., Feldt-Rasmussen U., Schiffmann R., Nicholls K. (2021). Long-term follow-up of renal function in patients treated with migalastat for Fabry disease. Mol. Genet. Metab. Rep..

[22] Pisani A., Wilson K.M., Batista J.L., Kantola I., Ortiz A., Politei J., Al-Shaar L., Maski M., Crespo A., Ponce E., Linhart A. (2024). Clinical outcomes in patients switching from agalsidase beta to migalastat: a Fabry Registry analysis. J. Inherit. Metab. Dis..

[23] Lenders M, Nordbeck P, Kurschat C, Karabul N, Kaufeld J, Hennermann JB, Patten M, Cybulla M, Müntze J, Üçeyler N, Liu D, Das AM, Sommer C, Pogoda C, Reiermann S, Duning T, Gaedeke J, Stumpfe K, Blaschke D, Brand SM, Mann WA, Kampmann C, Muschol N, Canaan-Kühl S, Brand E. Treatment of Fabry's disease with migalastat: outcome from a prospective observational multicenter study (FAMOUS). Clin. Pharmacol. Ther. 2020;108(2):326–337. doi:10.1002/cpt.1832. Epub 2020 Apr 27. PMID: 32198894. 32198894

[24] Lenders M., Nordbeck P., Kurschat C., Eveslage M., Karabul N., Kaufeld J., Hennermann J.B., Patten M., Cybulla M., Müntze J., Üçeyler N., Liu D., Das A.M., Sommer C., Pogoda C., Reiermann S., Duning T., Gaedeke J., von Cossel K., Blaschke D., Brand S.M., Mann W.A., Kampmann C., Muschol N., Canaan-Kühl S., Brand E. (2022). Treatment of Fabry disease management with migalastat-outcome from a prospective 24 months observational multicenter study (FAMOUS). Eur. Heart J. Cardiovasc. Pharmacother..

[25] Camporeale A., Bandera F., Pieroni M., Pieruzzi F., Spada M., Bersano A., Econimo L., Lanzillo C., Rubino M., Mignani R., Motta I., Olivotto I., Tanini I., Valaperta R., Chow K., Baroni I., Boveri S., Graziani F., Pica S., Tondi L., Guazzi M., Lombardi M. (2023). Effect of migalastat on cArdiac InvOlvement in FabRry DiseAse: MAIORA study. J. Med. Genet..

[26] Kallish S., Camporeale A., Hopkin R.J., Jovanovic A., Nordbeck P., Veleva-Rotse B.O., Krusinska E., Torra R. (2025). Long-term efficacy of migalastat in females with Fabry disease. J. Med. Genet..

[27] Lenders M., Stappers F., Brand E. (2020). *In vitro* and *in vivo* amenability to migalastat in Fabry disease. Mol. Ther. Methods Clin. Dev..

[28] Lenders M., Menke E.R., Brand E. (2024). Biochemical amenability in Fabry patients under chaperone therapy-how and when to test?. BioDrugs.

[29] Bichet D.G., Hopkin R.J., Aguiar P., Allam S.R., Chien Y.H., Giugliani R., Kallish S., Kineen S., Lidove O., Niu D.M., Olivotto I., Politei J., Rakoski P., Torra R., Tøndel C., Hughes D.A. (2023). Consensus recommendations for the treatment and management of patients with Fabry disease on migalastat: a modified Delphi study. Front. Med..

[30] Oommen S., Zhou Y., Meiyappan M., Gurevich A., Qiu Y. (2019). Inter-assay variability influences migalastat amenability assessments among Fabry disease variants. Mol. Genet. Metab..

[31] Schiffmann R., Bichet D.G., Benjamin E., Wu X., Giugliani R. (2019). The migalastat GLP-HEK assay is the gold standard for determining amenability in patients with Fabry disease. Mol. Genet. Metab. Rep..

[32] Johnson F.K., Mudd P.N., DiMino T., Vosk J., Sitaraman S., Boudes P., France N., Barlow C. (2015). An open-label study to determine the pharmacokinetics and safety of migalastat HCl in subjects with impaired renal function and healthy subjects with normal renal function. Clin. Pharmacol. Drug Dev..

[33] Feldt-Rasmussen U., Hughes D., Sunder-Plassmann G., Shankar S., Nedd K., Olivotto I., Ortiz D., Ohashi T., Hamazaki T., Skuban N., Yu J., Barth J.A., Nicholls K. (2020). Long-term efficacy and safety of migalastat treatment in Fabry disease: 30-month results from the open-label extension of the randomized, phase 3 ATTRACT study. Mol. Genet. Metab..

[34] Majid H., Verma N., Bhandari S., Gupta S., Nidhi A. (2024). Systematic review on safety and efficacy of migalastat for the treatment of Fabry’s disease. Expert. Opin. Pharmacother..

