# Supplementary material for: Effectiveness and tolerability of migalastat in adult Fabry disease: A single regional centre experience
Source: Mol Genet Metab Rep. 2026 May 11;47:101316. doi: 10.1016/j.ymgmr.2026.101316 (PMC13191641; doi:10.1016/j.ymgmr.2026.101316)
Supplement: Supplementary material [file mmc1.docx]

Supplementary material

| **Variable** | **Total**  **(87)** | **Male**  **(55)** | **Female**  **(32)** | **p-Value** |
| --- | --- | --- | --- | --- |
| RASi (ACEi + ARB) | 21 (24.1) | 13 (23.6) | 8 (25) | 0.886 |
| Beta blockers | 19 (21.8) | 14 (25.5) | 5 (15.6) | 0.285 |
| Thiazide diuretics | 4 (4.6) | 1 (1.8) | 3 (9.4) | 0.105 |
| Calcium channel blocker (BP) | 7 (8) | 5 99.1) | 2 (6.3) | 0.639 |
| Calcium channel blocker (rate control) | 3 (3.4) | 2 (3.6) | 1 (3.1) | 0.900 |
| Nitrates | 2 (2.3) | 2 (3.6) | 0 | 0.275 |
| Anti-platelet (Aspirin + clopidogrel) | 14 (16.1) | 8 (14.5) | 6 (18.8) | 0.607 |
| Statin | 27 (31) | 15 (27.3) | 12 (37.5) | 0.320 |
| Metformin | 3 (3.4) | 3 (5.5) | 0 | 0.179 |
| Anticoagulants (Warfarin or DOAC) | 7 (8) | 5 (9.1) | 2 (6.3) | 0.639 |

**Table 1: Baseline use of medications among patients receiving migalastat, stratified by sex:** This table summarises the baseline use of medications in 87 patients treated with migalastat, stratified by sex (55 males and 32 females), showing no significant differences in use between sexes.

RASi – renin–angiotensin system inhibitors (ACEi or ARB); ACEi – angiotensin-converting enzyme inhibitor; ARB – angiotensin receptor blocker; DOAC – direct oral anticoagulant.

| **Patient** | **Sex** | **Age at diagnosis** | **Variant** | **TN/TS** | **Length of treatment** | **Outcome** | **Reason** | **LysoGb3**  **Baseline** | **LysoGb3**  **Follow-up** |
| --- | --- | --- | --- | --- | --- | --- | --- | --- | --- |
| 1 | M | 44 | c.644A>G / p.N215S | TS | 3 years | ERT | Worsening cardiac involvement  IVSd 0.9 to 1.8 | 8.7 | 3.7 |
| 2 | M | 8 | c.1235C>A / p.Thr412Asn | TS | <1 year | ERT | Increase in lysoGb3 | 6.9 | 23.8 |
| 3 | M | 22 | c.902G>A / p.R301Q | TN | 5 years | ERT | Worsening cardiac involvement IVSd 0.6-1.2 | 8.6 | 3.2 |
| 4 | M | 16 | c.695T>C / p.I232T | TN | 1 year | ERT | GI side effects | 14.7 | 5.9 |
| 5 | M | 23 | c.1066C>T/ p.R356W | TN | 1 year | ERT | Declining renal function eGFR <30 ml/min/1.73m^2^ | 4.3 | 2.9 |
| 6 | M | 67 | c.644A>G / p.N215S | TN | 3 years | Died | Significant cardiac involvement, heart failure | 19.3 | 5.1 |

**Table 2: Adverse events leading to treatment discontinuation or death in patients receiving migalastat:** This table summarises adverse outcomes in six patients who discontinued migalastat or died during follow-up. Events occurred across both TN and TS groups. Baseline LysoGb3 at beginning or treamtnet (either ERT or migalalstat) and follow up is after migalstat. Reported good compliance in all patients (apart form 4 who developed GI side effects).

|  | **Treatment naïve (N=47)** | | | **Treatment switch (N=36)** | | |
| --- | --- | --- | --- | --- | --- | --- |
| **Variables** | **Baseline** | **Follow-up** | **p-Value** | **Baseline** | **Follow-up** | **p-Value** |
| **Biochemical** |  |  |  |  |  |  |
| eGFR >90, ml/min/1.73m^2^ | 23/47 (48.9%) | 14/47 (29.8%) | **0.032** | 33/36 (91.7%) | 12/36 (33.3%) | **<0.001** |
| eGFR >60, ml/min/1.73m^2^ | 39/47 (83.0%) | 34/47 (72.3%) | 0.106 | 36/36 (100%) | 34/36 (94.4%) | 0.531 |
| uPCR, mg/mmol | 12 (7-22) | 13 (7.5-29.25) | 0.343 | 10.1 (7.0-16.0) | 13.0 (6.0-34.0) | 0.266 |
| uACR, mg/mmol | 3.57 (1.21-13.95) | 3.66 (1.255-11.20) | 1.000 | 1.73 (0.89-4.38) | 2.53 (1.11-17.20) | 0.198 |
| **Cardiovascular** |  |  |  |  |  |  |
| PR interval (ms) | 156 (146-173.5) | 167 (146-190) | 0.057 | 160 (138-176) | 163 (148-190) | 0.092 |
| QTc interval (ms) | 435 (410.0-454.5) | 446 (416-468) | 0.292 | 418.5 (397.8-434.4) | 447.0 (423-470.5) | **<0.001** |
| Systolic BP, mm Hg | 133.5 (121.0-148.0) | 132.5 (119.0-151.0) | 0.382 | 136 (120-145) | 132 (120-140) | 0.071 |
| Diastolic BP, mm Hg | 79 (72-89) | 82 (74-95) | 0.272 | 80 (74-87) | 82 (76-90) | 0.052 |
| Intraventricular septum at end-diastole (IVSd) (cm) | 1.3 (1-1.6) | 1.4 (1.2-1.68) | 0.195 | 1.3 (1.0-1.6) | 1.4 (1.3-1.7) | 0.075 |
| LVM (g) | 226.36 (177.3-325.56) | 245.75 (175.5-300.2) | 0.739 | 228.6 (189.4-303.4) | 231.9 (194.5-307) | 0.642 |
| LVMI (g/m^2^) | 107.92 (93.08-164.59) | 115.30 (93.60-161.80) | 0.736 | 125.21 (98.7-164.3) | 111.6 (93.3-135.9) | **0.009** |

**Table 3: Renal and cardiovascular parameters at baseline and follow-up in treatment-naïve and treatment-switch patients receiving migalastat, after exclusion of four patients carrying *GLA* variants classified as non-amenable (or not tested in the 2024 UK Galafold® amenability table).** Values are presented as n (%) or median (interquartile range). Comparisons between baseline and follow-up were performed using the Wilcoxon signed-rank test for continuous variables and the McNemars for categorical variables. A p-value <0.05 was considered statistically significant. Excluding non-amenable variants did not materially alter the findings. Renal function remained largely preserved in both groups, with stable proportions of patients maintaining eGFR >60 mL/min/1.73 m² and no progression to ESKD. Proteinuria and blood pressure showed no significant change. In treatment-naïve patients, cardiac electrical and structural measures remained stable. In treatment-switch patients, QTc prolongation persisted as statistically significant, and LVMI demonstrated a modest but significant reduction over time, while IVSd and LVM remained stable.

eGFR - estimated glomerular filtration rate; ESKD - end-stage kidney disease; IQR - interquartile range; IVSd - interventricular septal thickness at end-diastole; LVM - left ventricular mass; LVMI -left ventricular mass index; ms - milliseconds; QTc - corrected QT interval; uACR - urine albumin-to-creatinine ratio; uPCR - urine protein-to-creatinine ratio.

| **Variables** | **Baseline** | **Follow-up** | **p-Value** |
| --- | --- | --- | --- |
| PR interval (ms) | 161 (152-187) | 179 (150.5-196.5) | 0.384 |
| QTc interval (ms) | 430.5 (407-449.5) | 439 (399.5-469.5) | 0.711 |
| Intraventricular septum at end-diastole (IVSd) (cm) | 1.3 (1-1.7) | 1.4 (1.2-1.6) | 0.645 |
| LVM (g) | 254.6 (183.9-356.5) | 249.85 (179.3-319.9) | 0.502 |
| LVMI (g/m2) | 135.7 (96.1-167.9) | 112.7 (92.4-166.5) | 0.379 |

**Table 4: Cardiac parameters at baseline and follow-up in treatment-naïve patients with the p.N215S mutation receiving migalastat:** This table presents cardiac conduction and structural measurements in treatment-naïve patients with the p.N215S mutation at baseline and after 12 months of migalastat therapy.
